# AMPK and the Need to Breathe and Feed: What’s the Matter with Oxygen?

**DOI:** 10.3390/ijms21103518

**Published:** 2020-05-15

**Authors:** A. Mark Evans, D. Grahame Hardie

**Affiliations:** 1 Centre for Discovery Brain Sciences and Cardiovascular Science, Edinburgh Medical School, Hugh Robson Building, University of Edinburgh, Edinburgh EH8 9XD, UK; 2 Division of Cell Signalling and Immunology, School of Life Sciences, University of Dundee, Dow Street, Dundee DD1 5EH, UK; d.g.hardie@dundee.ac.uk

**Keywords:** AMPK, oxygen, altitude, hypoxic ventilatory response, hypoxic pulmonary vasoconstriction, feeding, seep-disordered breathing, pulmonary hypertension, acute respiratory distress syndrome, COVID-19

## Abstract

We live and to do so we must breathe and eat, so are we a combination of what we eat and breathe? Here, we will consider this question, and the role in this respect of the AMP-activated protein kinase (AMPK). Emerging evidence suggests that AMPK facilitates central and peripheral reflexes that coordinate breathing and oxygen supply, and contributes to the central regulation of feeding and food choice. We propose, therefore, that oxygen supply to the body is aligned with not only the quantity we eat, but also nutrient-based diet selection, and that the cell-specific expression pattern of AMPK subunit isoforms is critical to appropriate system alignment in this respect. Currently available information on how oxygen supply may be aligned with feeding and food choice, or vice versa, through our motivation to breathe and select particular nutrients is sparse, fragmented and lacks any integrated understanding. By addressing this, we aim to provide the foundations for a clinical perspective that reveals untapped potential, by highlighting how aberrant cell-specific changes in the expression of AMPK subunit isoforms could give rise, in part, to known associations between metabolic disease, such as obesity and type 2 diabetes, sleep-disordered breathing, pulmonary hypertension and acute respiratory distress syndrome.

## 1. Introduction

It is evident that we must breathe without serious interruption from birth to death, and we do so with such ease that it almost goes unnoticed, whether we cough, sneeze, snort or, from our partners’ perspective, snore. That is, until the onset of certain respiratory diseases, such as sleep-disordered breathing and pulmonary hypertension. These disorders may appear unrelated to each other and to be far removed from food choice and metabolic disease, but evidence of cross-associations is growing.

Breathing is coordinated by a sophisticated motor program, which develops in utero in order to coordinate lung ventilation after birth, and responds appropriately to changes in oxygen demand during such activities as exercise, sleep or ascent to altitude. The fundamental rhythmic patterns of ventilatory activity are coordinated by a respiratory central pattern generator (rCPG) through downstream motor outputs from the brainstem and spinal cord, in a similar way to any other type of locomotion or rhythmic behaviour [1]. This occurs independently of peripheral or higher (suprapontine) input, with every breath triggered by cyclical phases of inspiratory muscle contraction (diaphragm, external intercostals), and followed by passive expiration through relaxation of these muscles. In addition, active expiration may be engaged to increase ventilation through recruitment of expiratory muscles (the abdominals and internal intercostals) when the rate of metabolism requires more oxygen. Alternatively, at the end of inspiration, the process of post-inspiration may adapt to reduced requirements by lengthening contraction of the diaphragm and adduction of laryngeal muscles to slow expiratory airflow by increasing airway resistance. These are the principal components of normal breathing (eupnoea), during which the composition and phasic pattern of muscle recruitment/activity is state-dependent. Each component of this cycle is built into the rCPG, central to the activity of which is the pre-Botzinger Complex [2], a brainstem microcircuit from which the rhythm of inspiration originates. In the present context, it is important to note that breathing patterns exhibit acute adaptation when, for example, metabolism increases, and also adapts on longer timescales during growth and maturation, pregnancy, ageing, disease and injury. 

**Figure 1:**
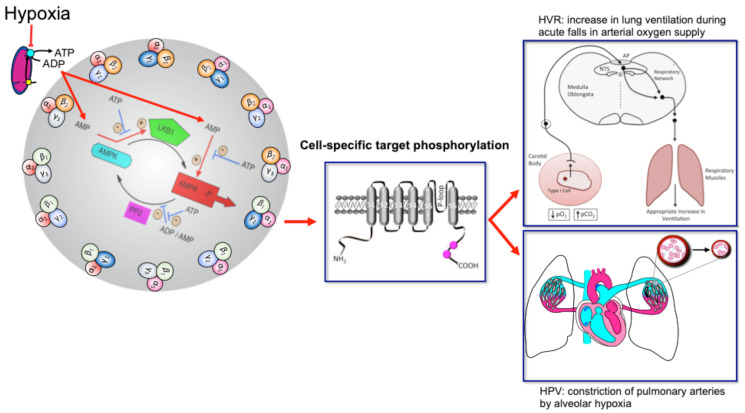
Turn the AMP up to 11 and breathe. Schematic shows the mechanism by which increases in the cellular AMP:ATP and ADP:ATP (adenosine monophosphate and diphosphate to adenosine triphosphate) ratios may activate up to 12 AMPK heterotrimeric subunit combinations, leading to target phosphorylation and induction of the hypoxic ventilatory response (HVR) and hypoxic pulmonary vasoconstriction (HPV). Red arrows indicate pathways leading to AMPK activation by hypoxia, flat heads indicate inhibition, arrow heads indicate activation. Blue arrows with flat heads indicate inhibitory pathways that contribute to the regulation of AMPK activity. Black arrows indicate direction of flow for subordinate pathways. Created by Evans.

Perhaps most important of all is our ability to adapt to deficits in oxygen supply, the most vital elixir of life. This is evident from birth, when hypoxia triggers the first breath due, in part, to the hypoxic ventilatory response (HVR; Figure 1). This response increases ventilatory drive through rCPG, and thus triggers subsequent adaptation to extrauterine life, through maturation of the carotid bodies, our primary peripheral chemoreceptors that drive HVR [3,4,5], and the airways, alveoli and pulmonary vasculature of the lungs that enable optimal gaseous exchange [6]. Gaseous exchange at our lungs is aided by another reflex response to falls in alveolar oxygen availability [7], namely hypoxic pulmonary vasoconstriction (HPV; Figure 1). HPV is a local response mediated by mechanisms intrinsic to the smooth muscles and endothelia of the pulmonary blood vessels, which aids ventilation-perfusion matching, by diverting blood flow through the path of least resistance, from oxygen-deprived to oxygen-rich areas of the lung. By contrast, systemic arteries dilate in response to hypoxia in order to meet the metabolic needs of the tissues they supply [8,9]. 

The mechanisms of hypoxia-response coupling in those specialised cells that coordinate cardiorespiratory reflex responses to falls in oxygen availability remain keenly debated topics, nowhere more so than with respect to HVR and HPV. However, emerging evidence now strongly suggests that the AMP-activated protein kinase (AMPK) might facilitate both HVR and HPV [10]. This hypothesis [11,12] was built on prior observations that mitochondria of oxygen-sensing cells were exquisitely sensitive to hypoxia, and the proposal that this was due to the selective expression in these cells of a form of cytochrome C oxidase (COX) that was uniquely sensitive to changing *P*O_2_ within the physiological range [13,14]. Support for this has been provided not only by the use of mitochondrial inhibitors [15,16,17,18,19,20], but by gene deletion strategies that examined the role of two nuclear encoded atypical subunits of the mitochondrial electron transport chain: (i) *NDUFA4L2* encoding NADH dehydrogenase [ubiquinone] 1 alpha subcomplex 4-like 2 (NDUFA4L2) [21]; and (ii) *COX4I2* encoding cytochrome c oxidase subunit 4 isoform 2 (COX4I2) [22,23]. NDUFA4L2 is a subunit of complex I, which transfers electrons from NADH to ubiquinone, while COX4I2 is a subunit of cytochrome c oxidase, which catalyses the transfer of electrons from cytochrome c to oxygen. NDUFA4L2 and COX4I2 are constitutively expressed under normoxia not only by oxygen-sensing type I cells of the carotid body [24], but also by pulmonary arterial myocytes [25,26]. In most other cell types NDUFA4L2 and COX4I2 expression is ordinarily low, although their expression may be increased during prolonged hypoxia [22,23]. Accordingly, carotid body type I cell responsiveness to acute hypoxia and acute HVR are abolished in mice by conditional deletion of *Cox4I2* in tyrosine hydroxylase expressing catecholaminergic cells [27], while HPV is occluded in isolated, ventilated and perfused lungs from Cox4I2 knockout mice [28]. Therefore, these atypical nuclear encoded subunits not only represent a further distinguishing feature of oxygen-sensing cells, but, at least in the case of COX4I2, appear to be critically important for hypoxia-response coupling within the physiological range of *P*O_2_.

Modulation of the properties of COX by COX4I2 is likely critical to hypoxia-response coupling in these highly specialised cells, because COX4I2 lowers the oxygen affinity of COX [29] and removes the inhibition by ATP that is normally conferred by COX4I1 [23,30]. In oxygen-sensing cells, therefore, COX4I2 expression may facilitate rapid decreases in ATP production as oxygen availability falls within the physiological range (~100 mmHg to ~20 mmHg) [31], in part because the rate of mitochondrial oxidative phosphorylation will not increase as ATP levels decline [22,25,30,32]. COX4I2 may thus accelerate increases in adenosine diphosphate (ADP):adenosine triphosphate (ATP) ratios, which would in turn increase adenosine monophosphate (AMP):ATP ratios via the adenylate kinase reaction [33,34], leading ultimately to concomitant activation of AMPK [35,36]. 

Consistent with this, all agents that inhibit mitochondrial oxidative phosphorylation activate AMPK in an AMP-dependent manner [35,36]. Moreover, there is now a growing realisation that AMPK is capable of phosphorylating targets outside of those canonical pathways by which it regulates cell-autonomous metabolic homeostasis [37,38,39,40,41,42,43,44,45,46]. In this way, it appears that, during evolution, the role of AMPK in regulating metabolic homeostasis has been extended through natural selection to support system-level control of substrate supply (e.g., of oxygen, glucose and fatty acids) in order to maintain energy (ATP) homeostasis across the whole body [11].

## 2. The AMP-Activated Protein Kinase and Cellular Metabolic Homeostasis

AMPK is a cellular energy sensor that was originally thought to maintain energy homeostasis in a cell autonomous manner. It exists as heterotrimers comprising one of two catalytic α subunits, in combination with one each of the two β and three γ regulatory subunits; together these may form at least 12 different heterotrimeric subunit combinations (Figure 1) [47]. Evidence is now emerging to suggest that different subunit combinations are expressed in different cell types, and that each combination may exhibit differential sensitivities to activation by AMP and ADP and thus metabolic stresses [48], and may selectively phosphorylate and regulate a different spectrum of target proteins due to differing subcellular locations [49,50]. 

There are four potential nucleotide binding sites formed by the cystathione-beta-synthase domain (CBS) repeats on the γ subunit, although only the CBS1, CBS3 and CBS4 sites may actually be utilised [51,52]. Binding of AMP to the CBS3 site causes a 10-fold increase in AMPK activity by allosteric activation, and also promotes further activation of up to 100-fold by promoting phosphorylation, and inhibiting dephosphorylation, at Thr172 within the activation loop of the α subunit; all of these effects are opposed by ATP binding [53,54]. Binding of ADP may also mimic the effects of AMP on Thr172 phosphorylation [55] and dephosphorylation [56], although allosteric action is only triggered by AMP. Thr172 is primarily phosphorylated by the tumour suppressor kinase LKB1, which appears to be constitutively active [57], but phosphorylates AMPK more rapidly when AMP or ADP is bound to the γ subunit. There is also an alternative mechanism involving the calmodulin-dependent protein kinase CaMKK2 [58,59], which phosphorylates Thr172 and thus activates AMPK in a Ca^2+^-dependent, AMP-independent manner [47,60,61,62]. Contrary to previous proposals [63,64], more recent evidence supports the view that AMPK is not directly activated by reactive oxygen species (ROS), although ROS can activate AMPK indirectly by inhibiting mitochondrial oxidative phosphorylation and increasing cellular AMP:ATP and ADP:ATP ratios [35,36]. Once activated, the classical action of AMPK is to phosphorylate targets that switch off non-essential anabolic processes that consume ATP and switch on catabolic pathways that generate ATP [62,65], thereby compensating for deficits in ATP supply via, for example, reductions in mitochondrial oxidative-phosphorylation. 

## 3. Phosphorylation by AMPK of Non-Metabolic Targets That Modulate Physiological Systems

It is becoming evident that AMPK also phosphorylates and thus modulates a variety of targets that fall outside of its originally proposed role in the maintenance of metabolic homeostasis [10,11,66,67,68]. For example, AMPK not only phosphorylates and thus *inactivates* the pore-forming α subunits of multiple Ca^2+^-activated potassium channels (K_Ca_1.1 and K_Ca_3.1) [45,69], the voltage-gated potassium channel K_V_1.5 [37,38,39] and the ATP-inhibited K_ATP_ channel (Kir6.2) [70], but also phosphorylates and *activates* the α subunit of the voltage-gated potassium channel Kv2.1 [46]. Evidence is also now emerging that AMPK may directly phosphorylate and regulate: (i) enzymes involved in the biosynthesis of specific transmitters [40,41,42]; (ii) receptors for neurotransmitters [43]; and (3) pumps and transporters [44,71]. In short, its downstream targets provide the necessary “toolkit” via which AMPK may modulate whole body energy homeostasis, through central control of system-specific outputs [11] that may coordinate breathing, feeding and, for that matter, food choice.

## 4. AMPK Aids HPV and Thus Gaseous Exchange at The Lungs 

Investigations into the role of AMPK in oxygen supply began with consideration of its role in HPV [12,72]. HPV is triggered by airway and/or alveolar hypoxia [7] rather than by vascular hypoxaemia [73]. HPV occurs through the constriction of pre-capillary resistance arteries within the pulmonary circulation, in a manner coordinated by signalling pathways that are intrinsic to their smooth muscles and endothelial cells [74,75,76], independently of blood-borne mediators or the autonomic nervous system [77,78]. The initiation phase of acute HPV is primarily driven by smooth muscle constriction [74], with a threshold *P*O_2_ ≈80 mmHg [79]. In addition to COX4I2 expression, AMPK-α1 levels are markedly higher in pulmonary compared to systemic arterial smooth muscles [72]. These factors may contribute to the cell-specific engineering that provides for pulmonary vascular constriction in response to hypoxia, and thus ventilation-perfusion matching, rather than arterial dilation in response to hypoxia by which the systemic vasculature ensures that oxygen availability meets the needs of the organs and tissues supplied by those vessels [8]. In an evolutionary context, it is intriguing to note that the lungs represent a relatively late adaptation [80,81,82], the transition to which is still evident in the metamorphosis of gill-breathing tadpoles to lung-assisted, air-breathing adult amphibians [83].

Exposure of pulmonary arterial smooth muscles to physiological hypoxia increases cellular AMP:ATP and ADP:ATP ratios, leading to concomitant AMPK activation and consequent phosphorylation of acetyl-CoA carboxylase (ACC; a well-validated marker for AMPK activation) [72]. Accordingly, relatively mild mitochondrial inhibition using the biguanide phenformin [84,85], such as hypoxia, increased cellular NAD(P)H auto-fluorescence, activated AMPK and increased ACC phosphorylation [72]. By contrast, AICA riboside, which is taken up into cells and metabolised to the AMP mimetic ZMP (AICA riboside monophosphate) [86], activated AMPK and increased ACC phosphorylation without affecting NAD(P)H auto-fluorescence [72]. 

Critically, AMPK activation by AICA riboside evoked a slow, sustained and reversible constriction of pulmonary artery rings, with strikingly similar characteristics when compared to the sustained phase of HPV, namely: (1) a requirement for smooth muscle SR Ca^2+^ release via ryanodine receptors that is retained, albeit attenuated, after removal of extracellular Ca^2+^; (2) Ca^2+^ influx into and vasoconstrictor release from the pulmonary artery endothelium [72]. Consistent with these findings, HPV was also inhibited by the non-selective AMPK antagonist compound C [87]. However, any action of this agent should be considered with caution, because in a screen of 70 protein kinases, it was shown to inhibit several other kinases more potently than AMPK [88]. 

In pulmonary arterial smooth muscles, the first functionally relevant and hypoxia-responsive molecular target to be identified downstream of mitochondria was the voltage-gated ion channel K_V_1.5, which is rapidly and reversibly inhibited by hypoxia [16,89,90]. The precise role of K_V_1.5 inhibition in acute HPV remains open to debate. Nevertheless, it provides a well-defined molecular target, the regulation of which by AMPK has been explored using new and more selective small molecule activators of AMPK, namely A769662 [91,92] and Compound 13 [93]. Each of these agents triggered AMPK-dependent phosphorylation of recombinant K_V_1.5 channel α subunits and inhibited potassium currents carried by these channels when expressed in HEK293 cells, while recombinant activated human AMPK phosphorylated K_V_1.5 α subunits in cell-free assays, at serine residues S559 and S592 [39,50]. A non-phosphorylatable K_V_1.5 mutation (S559A) virtually abolished inhibition by Compound 13 of K_V_1.5 currents, albeit with a modest impact on overall phosphorylation. By contrast, an S592A mutation reduced phosphorylation to a larger extent, but only partially blocked current inhibition, perhaps indicating cooperativity of function between S559 and S592 [50]. More significantly still, A769662 and Compound 13 not only inhibited K_V_ currents in acutely isolated pulmonary arterial smooth muscle cells, but occluded further inhibition of these potassium currents by hypoxia and inhibitors of mitochondrial oxidative phosphorylation [39]. Consistent with the action of compound 13, which is an AMPK-α1-selective agonist [93], conditional deletion of AMPK-α1, but not AMPK-α2, in smooth muscles almost abolished inhibition of K_V_1.5 channel currents in pulmonary arterial myocytes by hypoxia and mitochondrial inhibitors [50]. By contrast, however, intracellular dialysis of AMPK heterotrimers containing either AMPK-α1 or AMPK-α2 inhibited K_V_1.5 in rat pulmonary arterial myocytes [39], adding further weight to the argument that target specificity is determined by the anchoring of endogenous AMPK heterotrimers proximal to their targets, rather than by the intrinsic target specificity of any given subunit combination. The AMPK isoforms used in these latter studies were bacterially expressed α1β2γ1 or α2β2γ1 complexes that had been irreversibly activated by thiophosphorylation of Thr172 (thiophosphorylated serine/threonine residues are completely resistant to dephosphorylation), while the controls had mutations in their kinase domain that abolished kinase activity [45].

Conclusive evidence of a role for the LKB1-AMPK signalling pathways in HPV in vivo was then provided by gene deletion strategies in combination with spectral Doppler ultrasound, which demonstrated that HPV is markedly attenuated by global knockdown of the gene encoding LKB1 (*Stk11*), and virtually abolished by conditional deletion of AMPK-α1 in smooth muscles [50]. By contrast, HPV remained unaffected in mice with global CaMKK2 deletion [50]. As yet, however, the precise mechanisms by which AMPK mediates SR calcium release through ryanodine receptors of pulmonary arterial myocytes remains to be determined, while the proposed requirement for endothelial AMPK in acute HPV has yet to be confirmed.

Adding to this, it is evident that divergent LKB1-AMPK signalling pathways might coordinate HPV and pulmonary vascular metabolism downstream of mitochondria. Thus, deletion in smooth muscles of AMPK-α1, but not AMPK-α2, blocked the induction of HPV during mild hypoxia, indicating that there is no redundancy of function in this respect between AMPK catalytic subunit isoforms. By contrast, deletion of AMPK-α1 and AMPK-α2, respectively, attenuated HPV during exposure of mice to severe alveolar hypoxia. This indicates, perhaps, that AMPK-α2-containing heterotrimers are required in some specific way to coordinate ATP supply in support of HPV, rather than to regulate smooth muscle constriction *per se*. 

The finding that AMPK-α1 is critical to both K_V_1.5 inhibition and HPV is all the more intriguing, given that SNPs in the *PRKAA1* gene (encoding AMPK-α1) have been identified in native Andean populations that live at and are adapted to high altitude [94], and exhibit attenuated HPV [95].

## 5. AMPK and Central Neural Control Mechanisms

By acting centrally, AMPK may contribute yet wider system-specific control by influencing neural circuit mechanisms that serve to balance breathing, energy intake and energy expenditure. As mentioned above and exemplified by our studies on HPV, AMPK may achieve this via cell-specific expression not only of different AMPK subunit isoforms, but also of unique sets of receptors for hormones and neurotransmitters, and ion channels. In this way AMPK may confer, according to the location, system-specific differences in sensitivities to metabolic stresses, such as oxygen or glucose deprivation, or to hormones and neurotransmitters that activate AMPK via the CaMKK2 pathway. 

**Figure 2:**
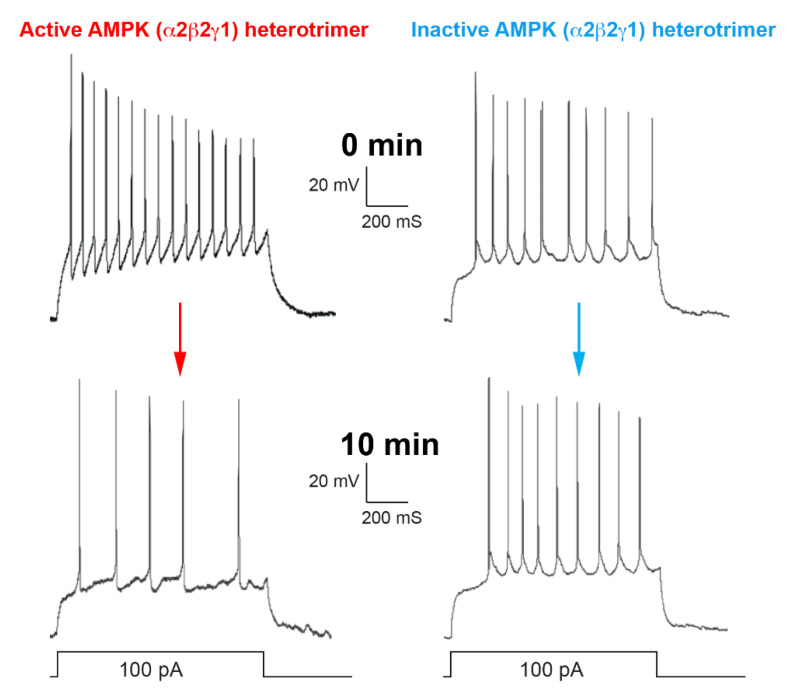
When the clock strikes 12 well cool off then. Active or inactive complexes of AMPK were applied intracellularly by dialysis from a patch pipette, and recordings taken at 0 min and 10 min after entering the whole-cell configuration. Action potentials were triggered by current injection. Created by Evans.

One way in which AMPK may regulate central neural control mechanisms is illustrated by our most detailed study on the regulation by AMPK of another ion channel, namely K_V_2.1. Similar to K_V_1.5, AMPK phosphorylates K_V_2.1 in cell-free assays and in intact cells at two sites (Ser440 and Ser537) within the C-terminal cytoplasmic tail [46]. In HEK-293 cells stably expressing K_V_2.1, AMPK activation using A-769662 caused hyperpolarising shifts in the current–voltage relationship for channel activation and inactivation, which were almost abolished by single (S440A) and completely abolished by double (S440A/S537A) phosphorylation-resistant mutations. In cells expressing wild type K_V_2.1, channel activation was also observed upon the intracellular administration of activated, thiophosphorylated AMPK (α2β2γ1), but not an inactive control [46]. K_V_2.1 is a voltage-gated, delayed rectifier potassium channel. Because of its relatively slow opening and closing in response to depolarisation, it is not thought to be involved in repolarising neurons after single action potentials, but instead to contribute to adjustments in the firing frequency of action potentials by raising the threshold membrane potential that must be reached between successive activations of the voltage-gated Na^+^ channels that determine action potential frequency. Accordingly, treatment of primary rat hippocampal neurons in culture with A-769662 caused hyperpolarising shifts in gating qualitatively similar to those observed in HEK-293 cells expressing K_V_2.1. That this effect was mediated by K_V_2.1 was indicated by its blockade when an anti-K_V_2.1 antibody was applied intracellularly via the patch pipette. Moreover, intracellular administration of active, thiophosphorylated α2β2γ1 complexes reduced the firing of action potentials in the neurons as predicted, whereas inactive control complexes had no effect (Figure 2). Therefore, AMPK not only regulates metabolic homeostasis of neurons, but also neuronal firing frequency. 

Kv2.1 is widely expressed in the central nervous system, particularly in pyramidal neurons in the hippocampus and cortex [96]. By contrast, the expression of Kv1.5, which is inhibited rather than activated by AMPK [39,50], appears to be restricted to the caudate putamen, the granule layer of the cerebellum, trigeminal sensory ganglia, pituitary gland and olfactory system (olfactory bulb, trigeminal terminal nerves and nasal vomeronasal organ) [97,98,99]. This highlights well the capacity for cell- and system-specific modulation of neural circuit mechanisms by AMPK.

The firing of action potentials, together with downstream postsynaptic events, are estimated to account for up to 80% of all energy turnover in the grey matter of rodent brain [100]. Since K_V_2.1 is widely expressed, its activation by AMPK could be a cell-autonomous mechanism to conserve energy by reducing membrane excitability and firing of action potentials in response to energy stress. It is interesting to speculate that this mechanism might also contribute during sleep, when breathing slows and central energy reserves are replenished. Indeed, knockdown of AMPK in the central nervous system of the fruit fly *Drosophila melanogaster* led to disturbances in sleep patterns and to poorer recovery from sleep deprivation [101]. 

During drowsiness and light non-rapid eye movement sleep, a moderate decrease in the oscillating breathing pattern occurs, with significant variation in periodicity of ventilation due to, in part, instability of central respiratory drive and blunted HVR. By contrast, in deep non-rapid eye movement sleep, the pattern of breathing is more regular, although the tendency to hypoventilate increases. This hypoventilation seems to be the result of both decreased ventilatory drive and increased upper airway resistance. Rapid eye movement (REM) sleep is characterised by more irregular and yet shallower breathing, synchronous with REM bursts [102]. During the sleep cycle, the carotid bodies and downstream chemo-responsive respiratory networks of the brain continue to monitor oxygen and carbon dioxide levels, and coordinate appropriate adjustments to breathing, sympathetic tone and blood pressure. Periodic hypoxia and hypercapnia may result from brief periods of breathing cessation (apnoea), which occur in normal subjects during sleep, but become more frequent and prolonged in patients suffering from sleep-disordered breathing, due either to obstruction of the upper airway (obstructive sleep apnoea) or defective respiratory rhythm generation by the central nervous system (central sleep apnoea). Apnoea leads to increased respiratory drive through activation of chemosensory networks, and subsequent arousal from sleep [103,104,105].

This brings us neatly to cell- and circuit-specific functions of the nervous system through which AMPK may contribute to the regulation of breathing, energy supply and energy expenditure during wakefulness.

## 6. AMPK, HVR and Oxygen Supply

As mentioned previously, AMPK is intimately coupled to mitochondrial metabolism through sensing of cellular AMP:ATP and ADP:ATP ratios, and mitochondria of oxygen-sensing cells appear to be uniquely sensitive to changes in oxygen supply because of their cell-specific expression of two nuclear-encoded, atypical subunits of the mitochondrial electron transport chain, NDUFA4L2 [21] and COX4I2 [22,23,29]. Of these, gene deletion strategies have confirmed that COX4I2 is critical to the activation of both oxygen-sensing carotid body type I cells and acute HVR [27].

Consistent with a role for AMPK downstream of mitochondria, HVR is markedly attenuated by conditional deletion of LKB1 [106] or AMPK-α1, and more severely still by the dual deletion of both AMPK-α1 and -α2, using Cre recombinase expression from the tyrosine hydroxylase promoter [107]. In fact, exposure of these mice to hypoxia triggers hypo-ventilation and apnoea, rather than hyper-ventilation as in the wild type [106,107]. Note that catecholaminergic cells expressing tyrosine hydroxylase span the entire hypoxia-responsive respiratory network, including therein type I cells of carotid bodies and brainstem neurons that lie downstream. 

**Figure 3:**
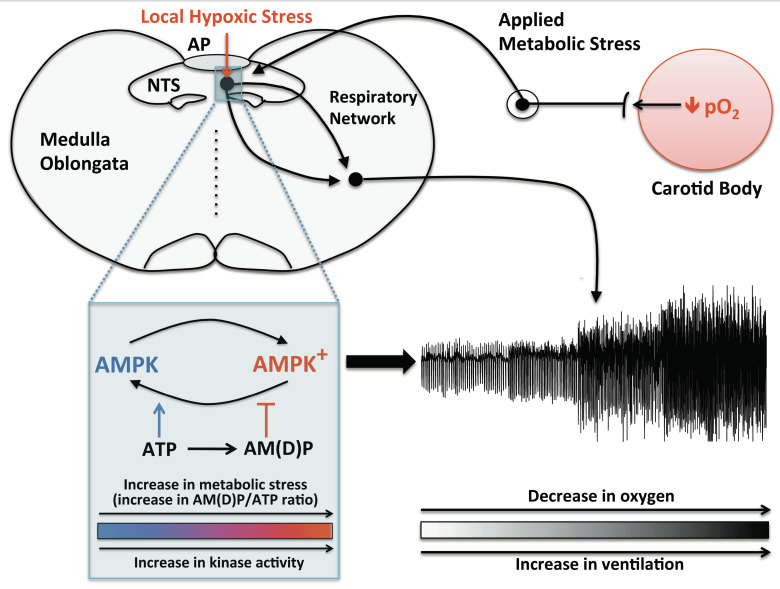
One, two, three o’clock, four o’clock breathe. Schematic describing the new hypothesis on the regulation by AMPK of the hypoxic ventilatory response, through the integration of local and applied metabolic stresses. AP = area postrema; NTS = nucleus tractus solitarius. From [107], created by the Evans laboratory and redrawn by Evans here.

The primary peripheral arterial chemoreceptors of mammals are the carotid bodies [108], of which type I cells represent the archetypal oxygen-sensing cells [109]. The general consensus has been that it is the afferent input responses of carotid bodies that deliver the entire ventilatory response to falls in arterial *P*O_2_ [5,110]. Challenging this, however, AMPK deletion attenuated HVR during mild and severe hypoxia without affecting these afferent input responses. This is consistent with findings that two compounds that activate AMPK via different mechanisms, i.e., AICA riboside [86] and A-769662 [91,92], do not precisely mimic the effects of hypoxia or induce pronounced activation of carotid body type I cells [111]. Thus, peripheral chemosensors may not be the sole arbiters of HVR. This has also been suggested by investigations on the evolution of ventilatory control systems. Intriguingly, oxygen-sensing and a component of HVR occurs at the level of the caudal brainstem in amphibians, in which both the location and influence of the primary peripheral chemosensors change during metamorphosis from gill-breathing tadpole to lung-assisted, air-breathing adult [83]. It was proposed [112], therefore, that evolution periodically led to the reconfiguration of peripheral chemoreceptor inputs [83] about a common, ancestral sensor of hypoxia within the caudal brainstem (Figure 3), which effects signal integration and thus acts as the gatekeeper of respiratory adjustments during hypoxia. In short, HVR may be determined by the coordinated action of the carotid body and a wider hypoxia-responsive circuit within the brainstem [107,112,113,114].

Until recently, little emphasis has been placed on the role of hypoxia-sensing by the brainstem, perhaps because HVR is so effectively abolished by resection of the carotid sinus nerve in humans [115]. However, brainstem hypoxia induces HVR even when the brain is in receipt of normoxic carotid body afferent inputs [116], and does so by directly activating subsets of catecholaminergic neurons within the nucleus tractus solitarius (NTS) and rostral ventrolateral medulla (RVLM), which may support partial recovery of HVR in a variety of animal models [113]. Consistent with the effect of AMPK deletion on HVR, dysfunction of these catecholaminergic neurons precipitates hypoventilation and apnoea associated with Rett syndrome, which is exacerbated during hypoxia [117]. Moreover, it is evident that COX4I2 may, as in carotid body type I cells, be constitutively expressed by certain CNS neurons [30], rendering mitochondrial oxidative phosphorylation sensitive to falls in local *P*O_2_ within the physiological range. AMPK activation could thus be triggered in a specialised subset of brainstem neurons during hypoxia, to support the delivery of increased respiratory drive required to protect against hypoventilation and apnoea. Supporting this, examination of brainstem function in AMPK-α1/α2 knockout mice by functional magnetic resonance imaging identified reduced activation of discrete dorsal and ventral nuclei of the caudal brainstem, despite the fact that carotid body afferent input responses were retained [107]. The location of the dorsal nucleus aligns with areas of the NTS that are activated during hypoxia and represent the primary site of receipt of carotid body afferent inputs [113], and is therefore well placed for signal integration. Here, AMPK deletion may attenuate activation during hypoxia of A2 (noradrenergic/dopaminergic) neurons proximal to the midline and the area postrema [107,118]. Notably, A2 neurons provide afferent inputs to and determine, together with the carotid body, activation by hypoxia of A1/C1 neurons within the RVLM [119] and the rCPG [1,120], the position of which aligns well with the ventral active region identified by fMRI analysis [107]. Through these projections of the NTS, AMPK could thus support HVR [10,112] by either direct or indirect modulation of the rCPG [1,120], and perhaps by also aiding the coordination of functional hyperaemia [121]. 

Carotid body afferent discharge of AMPK knockout mice remains exquisitely sensitive to falls in *P*O_2_, and ventilatory responses to hypercapnia remain unaffected even during severe hypoxia. It is therefore unlikely that AMPK deficiency compromises the capacity during hypoxia either for activation of the peripheral carotid body type I cells or the brainstem catecholaminergic neurons that govern the ventilatory response to hypercapnia, exocytosis or effective delivery of increased respiratory drive [112]. Therefore, the mechanism by which AMPK supports HVR is most likely neurogenic and highly localised, since AMPK deficiency in smooth muscles does not affect HVR or systemic arterial blood pressure regulation during hypoxia, while the latter (but not HVR) remains unaltered following AMPK deletion in catecholaminergic neurons [122]. This presents us with another example of cell- and system-specific actions of AMPK.

Accepting the possibility of a role in signal integration at the NTS, the working hypothesis is that AMPK activation by the canonical, AMP/ADP-dependent pathway may provide the capacity for sensing local hypoxic stress and integrate this with afferent input responses. It seems plausible that within these specialised NTS neurons, AMPK could very effectively integrate decreased ATP supply due to local hypoxia with increased ATP usage consequent to afferent inputs from peripheral chemoreceptors. The firing frequency of action potentials within these NTS neurons could thus be modulated by AMPK in a manner proportional to the overall ATP demand in the receiving, activated neurons [10,112]. Neuronal energy supply and activity across the entire circuit, from NTS to rCPG, may in turn be supported by direct activation during hypoxia of lactate and/or ATP release from astrocytes throughout the NTS, RVLM and rCPG [114,123,124,125], to which AMPK may also contribute by balancing neuronal TCA cycle dynamics [126]. In this respect, it is notable that astrocytes also express COX4I2 [30], because this atypical subunit of the mitochondrial electron transport chain may not only confer acute sensitivity to physiological hypoxia, but also reduce the capacity for ATP supply via the TCA cycle in those cells that express it [29]. 

## 7. The Role of Hypothalamic AMPK in Regulating Appetite and Feeding Behaviour

**Figure 4:**
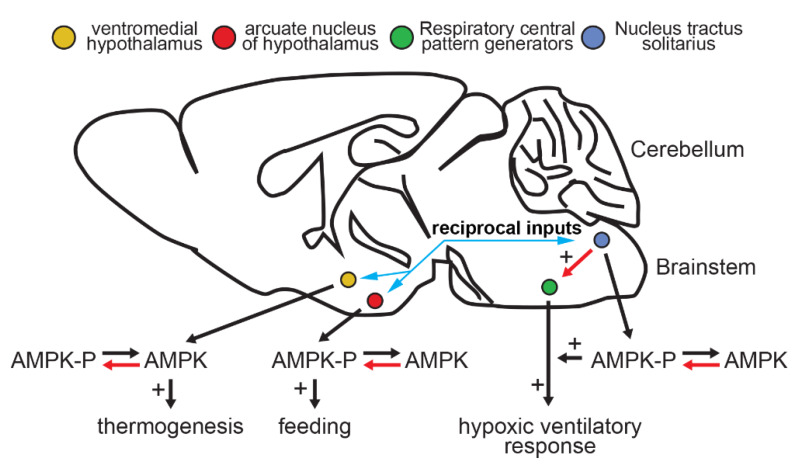
When the clock strikes four feed and breathe some more. The diagram shows a tracing of a sagittal section of a whole mouse brain, indicating anatomical locations where AMPK may regulate the neural control of appetite/feeding (arcuate nucleus), energy expenditure (ventromedial hypothalamus) and breathing nucleus tractus solitarius (NTS) + respiratory central pattern generators (rCPG). Red arrows indicate pathway to AMPK, or rCPG activation. Black arrows indicate pathways to increased breathing, feeding and thermogenesis. Blue arrows indicate reciprocal pathways of innervation between the NTS, arcuate nucleus and ventromedial hypothalamus. Created by Evans and Hardie.

Among the most well-established roles of AMPK in the nervous system is in the regulation of appetite and food intake, which highlights further system-specific functions (Figure 4). Feeding is known to be promoted by the stimulation of neurons located in the arcuate nucleus of the hypothalamus that express agouti-related protein (AGRP) and neuropeptide Y (NPY), and to be inhibited by neurons in the same anatomical location that express pro-opiomelanocortin (POMC) and cocaine-and-amphetamine-regulated transcript (CART). NPY/AgRP neurons increase food intake and decrease energy expenditure by antagonising POMC action on melanocortin receptors in neurons of the paraventricular nucleus (PVN). First indications of a role for AMPK in feeding behaviour came from findings that indicated it was activated in rat hypothalamus by treatment in vivo with the orexigenic hormone ghrelin, and inhibited by treatment with the anorexigenic hormone leptin [127]. Other orexigenic mediators, such as cannabinoids, were subsequently found to also activate AMPK [128]. Moreover, injection of the AMPK activator AICA riboside into the hypothalamus led to increases in food intake [127]. Although AICA riboside is now known to have many “off-target” (AMPK-independent) effects, the conclusion that its effects on feeding were AMPK-mediated was strengthened by findings that ectopic expression in mouse hypothalamus of inactive mutants of AMPK-α1 and -α2 (which lack kinase activity, but exert a dominant negative effect by competing with endogenous α subunits for binding to β and γ subunits) repressed food intake and body weight gain. Conversely, expression of an activated γ1 mutant (which behaves as if AMP is bound even when it is not) had the opposite effects. Moreover, leptin was found to decrease the activity of AMPK complexes containing α2, but not α1, in the hypothalamus [129]. The phenotypes of knocking out AMPK-α2 in AGRP/NPY and POMC/CART neurons, respectively, were also initially consistent with a role for AMPK in appetite control, with the former being lean while the latter were obese. However, in both cases the effects were rather modest and age-dependent, and there were no detectable changes in food intake in the AGRP/NPY knockouts, while the POMC/CART knockouts still responded normally to leptin treatment in terms of food intake and body weight, and to leptin and insulin (another anorexigenic hormone) in electrophysiological studies [130]. Interestingly, a proportion of both AGRP/NPY and POMC/CART neurons responded to glucose deprivation by hyperpolarisation and a consequent reduction in spike frequency, although in neither case was this evident when AMPK-α2 was knocked out in these neurons. Thus, although AMPK does not appear to be required for the response to leptin and insulin in these specific (AGRP/NPY or POMC/CART) neurons, it does seem to be required for glucose-sensing [130]. The latter point is interesting given the evidence that AMPK can sense glucose in other cells via a non-canonical mechanism [131].

The apparent lack of effect of knocking out AMPK in AGRP/NPY or POMC/CART neurons on food intake [130] may be because AMPK is required not in these neurons *per se*, but in other neurons acting immediately upstream or downstream. In one study, AGRP/NPY neurons were identified by their fluorescence in brain slices derived from transgenic mice expressing a fluorescent protein fused to NPY, and the activity of presynaptic neurons was assessed by measuring miniature excitatory postsynaptic currents (mEPCs) in these cells, in the presence of tetrodotoxin to suppress firing of action potentials [132]. Interestingly, treatment of brain slices from fed mice with the orexigenic hormone ghrelin increased mEPCs in the AGRP/NPY neurons to the level seen in fasted mice. Studies with pharmacological agents suggested that this was mediated by stimulation of the ghrelin receptor Ghsr1, which is coupled to intracellular release of Ca^2+^ via the G protein G_q_/G_11_. This would initiate a Ca^2+^-dependent activation of AMPK in the presynaptic neurons via the CaMKK2 pathway. There was already evidence of CaMKK2 involvement in the response to orexigenic signals, based on studies using the CaMKK2 inhibitor STO-609 and global mouse knockouts of CaMKK2 [133]. Therefore, although Yang et al. [132] relied heavily on different pharmacological agents, some of which might have off-target effects, their study provides a plausible model to explain the role of AMPK in feeding and appetite control in specific neurons of the hypothalamus. One proposal to explain the orexigenic effects of ghrelin in the hypothalamus, downstream of the CaMKK2-AMPK pathway, involves reductions in malonyl-CoA levels due to inhibition of acetyl-CoA carboxylase by AMPK, with consequent activation of carnitine-palmitoyl transferase-1C (CPT1C) [134] and increased levels of ceramides [135].

## 8. AMPK and Food Choice

The aforementioned findings are all the more intriguing, given recent work [136] that showed that expression of constitutively active AMPK in hypothalamic PVN neurons led to a preference for carbohydrate over fat in food choice of mice, through activation by AMPK of CPT1C, which promotes mitochondrial β-oxidation of fatty acids, within a subset of corticotrophin-releasing hormone-positive neurons in the rostral region of the PVN. It is possible that this affords a coordinating link between adaptive changes to feeding and food choice and ventilatory oxygen supply, given the evident reciprocal inputs between PVN and NTS (Figure 4) that modify peripheral and central cardiorespiratory reflex responses during hypoxia [137,138,139,140].

## 9. Hypothalamic AMPK and Energy Expenditure through Thermogenesis

In another region of the hypothalamus, the ventromedial nucleus (VMH), AMPK appears to be involved in the regulation of peripheral energy expenditure rather than energy intake, by regulating the firing of sympathetic nerves that stimulate fatty acid oxidation, and hence, heat production (thermogenesis) in brown adipose tissue. Thus, direct administration to VMH by intra-cerebroventricular injection of the female sex hormone estradiol [141], the thyroid hormone T3 [142], the GLP-1 receptor agonist liraglutide [143] or Bone Morphogenetic Protein-8B (BMP8B) [144,145] all reduced phosphorylation of AMPK in VMH and increased thermogenesis. In the cases of estradiol and T3 this was associated with increases in the activity of sympathetic nerves and weight loss. Moreover, injection of adenoviruses expressing an activated mutant of AMPK reduced the weight loss associated with either hormone treatment [141,142]. The effects of T3 in VMH were also mediated in part by stimulation of the vagus nerve to promote lipogenesis in the liver [146]. This effect on thermogenesis, and hence energy expenditure, appears to be mediated by inhibition of AMPK complexes containing the α1 isoform in neurons of the VMH expressing Steroidogenic Factor-1 (SF1), because mice with a specific knockout of AMPK-α1 in these neurons recapitulated the effect of central T3 [146]. These mice displayed increased thermogenesis and were resistant to diet-induced obesity [147]. Adding to this, a recent study [148] has highlighted downstream effects of AMPK. Briefly, chronic genetic activation of AMPK in mice via a D316 mutation in the γ1 subunit was shown to lead to the emergence, when placed on a high fat but not normal chow diet, of a population of subcutaneous white adipocytes with small, multilocular lipid droplets. A reduction in body weight and a decrease in fat mass but not lean mass was observed in these mice when switched to the high fat diet, alongside improved insulin sensitivity in the absence of any difference in glucose tolerance. Most interestingly, in the context of the present review, food intake was not significantly different on the high fat diet, but substantial increases in white adipose tissue oxygen consumption rate were associated with active thermogenesis. Moreover, while there were no differences in expression of PPAR-gamma co-activator 1 α (Pgc1α) or components of the mitochondrial electron-transport chain when these mice were fed a normal chow diet, increased expression of Pgc1α and thus mitochondrial proteins occurred when they were fed a high fat diet, consistent with browning of white adipocytes. Note that these findings provide evidence of cell- and context-specific changes in mitochondrial metabolism, and thus respiratory capacity, consequent with AMPK deficiency, and highlight the therapeutic potential for the treatment of obesity through increased whole-body energy expenditure.

Interestingly, while AMPK deficiency in sympathetic (catecholaminergic) neurons might increase thermogenesis and weight loss, and attenuate HVR, it does not appear to markedly impact systemic arterial blood pressure regulation during normoxia or hypoxia, which is determined in great part by increased sympathetic outflow [122]. The only way to reconcile this is if pathways leading to sympathetic control of thermogenesis differ from those involved in control of blood pressure, which is perhaps yet another example of cell- and system-specific outputs.

## 10. Regulation of Oxygen Supply, Feeding and Food Choice during Adaptive Acclimation to Hypoxia at Altitude

As one might expect, adaptive thermogenesis is triggered by cold both at sea level and altitude, and this is partly governed by those sympathetic pathways described above, which will also impact breathing, feeding and food choice, other than through energy expenditure pathways that may promote weight loss indirectly (for review see [149]). Moreover, during acclimation to altitude it is evident that there are differences between acute and chronic adaptive changes to breathing, feeding and food choice. Intriguingly, longer-term adjustments to ventilatory patterning are accompanied by changes in food choice, without loss of appetite *per se*. 

It is well recognised that time-dependent augmentation of HVR and thus increased ventilation accompanies adaptation to altitude [113,150]. In this respect, a recent study has highlighted the role of carotid body activation and type I cell proliferative responses to sustained hypoxia during ventilatory acclimatisation, a process that is mediated by HIF-2α, and abrogated by its inhibition [151]. However, evidence suggests that ventilatory acclimatisation is, at least in part, also determined by adjustment of NTS afferent inputs to RVLM [152], where regulation of AMPK-α1 expression [107] and consequent adjustments to AMPK-dependent facilitation of HVR may come in to play [10,112]. Adding to this, HPV is normally attenuated during sustained chronic hypoxia [153] and acclimation at altitude [154]. This may serve to oppose the development of chronic hypoxic pulmonary hypertension, and our data strongly suggest that repression of AMPK-α1 expression may be relevant here too [50]. This draws us back once again to the SNPs identified in *PRKAA1* of Andean populations that have adapted to life under relative hypoxia at altitude [94]. Intriguingly, this ethnic group enter relative hypoventilation during exercise at altitude [95], and exhibit attenuated HPV with postnatal persistence of pulmonary hypertension [95]. In short, it seems plausible that cardiorespiratory adaptation to hypoxia at altitude may be driven by cell-specific changes in AMPK subunit expression and/or AMPK activity, which is generally considered to be governed independently of HIF1-α or HIF2-α. However, it is noteworthy that those signalling pathways regulated by AMPK and HIF1/2-α may indirectly influence each other in a cell-specific manner, delivering both opposing and cooperative outcomes depending on the context. For example, activation of either AMPK or HIF may increase glucose uptake, glycolytic flux and autophagy, and suppress protein translation. However, where mitochondrial biogenesis is concerned they oppose each other, AMPK promoting mitochondrial biogenesis, while, by contrast, HIF signalling under prolonged hypoxia generally acts to decrease mitochondrial mass (for detailed review see [155]). Therefore, AMPK and HIF may, each in part, deliver appropriate adjustments to whole-body metabolism in a cell- and thus system-specific manner.

Adding to this, a growing body of evidence suggests that during the process of adaptive acclimation, neuroendocrine systems that contribute to the control of feeding respond differentially to acute hypoxia and chronic hypoxia. Moreover, we know chemoreflex pathways that govern ventilation are not restricted to the medulla and pons, but extend to the mesencephalic and hypothalamic regions, and in doing so confer developmentally regulated relays between caudal and ventral hypothalamic neurons that control feeding and thermogenesis, and those medullary neurons involved in central afferent input responses to hypoxia [156,157,158]. Reciprocal inputs also project from the PVN to the NTS, and may contribute to the modulation of NTS neuronal activation, chemoreflex input responses and thus cardiorespiratory control during hypoxia [137,138,139,140,159]. 

This role of the PVN in supporting arterial oxygen supply by modulating respiratory responses to hypoxia through the NTS [139,140] and the reciprocal innervation between the NTS and PVN, highlights the possibility that feeding and the efficiency of energy utilisation by the body may rely on similar levels of reciprocity [160]. This may be achieved, at least in part, through the influence of these pathways on not only feeding, but also food choice during hypoxia when oxygen availability declines at altitude [161], and perhaps, therefore, when oxygen intake and utilisation by the body falls with age and disease. Supporting this, it has been noted that upon ascent to altitude plasma levels of the “satiety hormone” leptin may increase immediately, followed by delayed falls in the “hunger hormone” ghrelin; it is also interesting to note in this regard that gestational hypoxia can disrupt the neonatal leptin surge in such a way that hyperphagia and obesity are programmed for adult life [162]. Moreover, during ascent of adults to altitude this leptin surge is accompanied by a delayed increase in NPY levels, and decreased POMC in the arcuate nucleus after seven days at altitude [163,164], and this too may be critical to the regulation of feeding behaviour during acclimation. In these respects, however, the possible role of AMPK remains to be explored.

It is nevertheless notable that when humans that dwell at sea level are acutely exposed to hypoxia, they exhibit increased anaerobic glucose metabolism [165]. Moreover, after acclimation at altitude (4300 M) muscle fatty acid consumption decreases during rest and exercise, while glucose uptake increases during exercise [166]. This switch to carbohydrates, and reduced reliance on lactate, occurs after 3 weeks of altitude acclimatisation in males [167,168], and likely persists in long-term high-altitude residents [169]. The reasons for this seem clear, because use of carbohydrates (glucose, glycogen) as fuel, rather than free fatty acids or other lipids, can generate 25%–50% more ATP per mole of oxygen consumed [170]. Accordingly, a high carbohydrate diet has been reported to increase endurance for heavy work at altitude, and also ameliorates symptoms of acute mountain sickness [171]. 

The possibility of coordinate regulation of breathing, feeding and food choice is all the more intriguing in this context, when one considers that this metabolic shift to carbohydrates is evident not only in newcomers acclimatised to altitude, but also in adapted Andeans, Tibetans and Sherpa (for review see [169,172]), and is associated with less sympathetic and/or greater parasympathetic stimulation [173,174,175]. Moreover, even after descent to sea level altitude-adapted Andean (Quechua) and Sherpa males exhibit a greater reliance on carbohydrate metabolism and a 50%–60% gain in the production of ATP per mole of oxygen consumption relative to lowlanders, which persists during acute hypoxia and moderate exercise [176]. The possible role of *PRKAA1* expression in these processes remains to be determined in either lowlanders or Andeans. However, in adapted Tibetans, who exhibit no identified SNPs in *PRKAA1*, it has been established that lactate levels are positively associated with an adaptive *EPAS1* (HIF1α) haplotype (and perhaps *EGLN1* which encodes prolyl hydroxylase domain-containing protein 2 (PHD2)), which is consistent with decreased glucose oxidation, while changes to serum free fatty acids are associated with a *PPARA* haplotype (*PPARA* encodes the nuclear receptor protein PPARα, a regulator of fatty acid oxidation [177]). 

## 11. Pathological Links between AMPK, Oxygen Supply and Food Choice 

In the context of disorders related to feeding and food choice among lowlanders, we gain further traction, and further evidence of reciprocity with respect to respiratory control mechanisms. Pulmonary hypertension and sleep-disordered breathing are not only symptomatic of altitude sickness [150,178,179], but also of metabolic disease [104,180,181,182,183], with which cell-specific alterations in AMPK subunit expression patterns are known to occur [184,185]. Adding to this, inverse relationships between residence at altitude and the risk of diabetes [175] and obesity [186] have been noted. This has been attributed to the association between chronic hypoxia at high altitude and decreased serum glucose and insulin concentrations in humans [187,188]. Consistent with this, Tibetan highlanders have a relatively low prevalence of diabetes [189], although they also have a relatively low-calorie diet [190], and relatively low body weight [191].

It seems equally plausible, therefore, that AMPK isoform expression might be affected by gestational hypoxia or chronic intermittent hypoxia in adults. Aberrant, cell-specific changes to AMPK isoform expression patterns could thereby be triggered and contribute to the onset of obesity, type 2 diabetes [162,192,193] and sleep-disordered breathing [103,113] in later life. This possibility gains indirect support from our studies, because AMPK deletion in catecholaminergic cells not only attenuates HVR, but also confers a neonate-like HVR characterised by delayed hypoventilation and apnoea in adults exposed to hypoxia, but with preservation of hypoxic-hypercapnic ventilatory responses. In short, these mice exhibit HVR similar to that of pre-term infants that are susceptible to sudden infant death syndrome [107,194]. In short, AMPK may be necessary in some as yet unrecognised way for appropriate maturational shifts from neonatal to adult HVR patterning [113]. Supporting this, neonatal exposure to poikilocapnic intermittent hypoxia precipitates sleep-disordered breathing in later life, in part through consequential, life-long depression of HVR [113]. Moreover, as mentioned above, gestational hypoxia disrupts the neonatal leptin surge in such a way that hyperphagia and obesity are programmed for adult life [162].

When considering pulmonary hypertension, however, we meet a paradox. AMPK facilitates HPV and thus acute hypoxic pulmonary hypertension, yet appears to oppose chronic hypoxic pulmonary hypertension [42,195,196]. This is logical, because hypoxic pulmonary hypertension is considered to be a proliferative disease, which would be opposed by the activation of the AMPK-ULK1-mTORC1 pathway, through the induction of autophagy [197] and the inhibition of lipid, protein and rRNA biosynthesis [198]. Accordingly, AMPK activation using metformin [199] may ameliorate chronic hypoxic pulmonary hypertension [200], although significant AMPK-independent actions of metformin might impact cell metabolism and function here [201,202]. Adding to this, it has been suggested that pulmonary arteries from human patients with idiopathic pulmonary hypertension exhibit AMPK deficiency [42,196]. If this is the case, then the progression of pulmonary hypertension, whether idiopathic or hypoxic, must be driven in some way through cell- and system-specific repression of AMPK expression. Given that HPV is facilitated by AMPK [50], this could partly explain why HPV is attenuated during chronic hypoxia [153] and acclimation at altitude [154], should progression from acute to chronic hypoxic pulmonary hypertension result from excessive repression of AMPK expression in the pulmonary vasculature during sustained chronic hypoxia. If this proves to be the case, then AMPK repression could also contribute to idiopathic neonatal pulmonary hypertension, a hypertensive vasculopathy exclusive to the pulmonary vasculature of children that is triggered by brief post-natal exposure to hypoxia and maternal metabolic inputs in utero [6,203,204]. 

These possibilities again receive indirect support from studies on Andeans if, as suspected, cell-specific AMPK-α1 repression is delivered through identified SNPs in *PRKAA1* [94], because Andeans not only enter relative hypoventilation during exercise at altitude [95], but also exhibit attenuated HPV with postnatal persistence of pulmonary hypertension [95]. Adding to this, it is evident that some animals that ordinarily live at and are adapted to high altitude, such as Guinea-pigs, exhibit markedly attenuated HPV [205] and HVR [206], yet retain, albeit to lesser degrees, the capacity for remodelling within the pulmonary vasculature [205,207] and brainstem [208,209] during sustained chronic and chronic intermittent hypoxia. 

When considering metabolic and respiratory disease in the context of oxygen supply at altitude, it is of further intrigue to note that while there is evidence of increased susceptibility to some infections at high altitude [210], those living at and adapted to high altitude (Andeans and Tibetans) may be less likely to develop severe adverse respiratory dysfunction during other infections, such as SARS-CoV-2 (COVID-19) [211]. Given that the innate and adaptive immune responses are subject to modulation by hypoxia [212], it is significant that they can be modulated by AMPK, in addition to HIFs-α, in either a beneficial or detrimental way depending on the nature of infection [213]. Supporting this, certain RNA and DNA viruses may trigger time-dependent reductions and/or increases in AMPK activity to facilitate viral entry, adjust cellular metabolism in order to facilitate replication, and to combat innate host cell immunity [214]. By contrast, AMPK may oppose bacterial infection through the induction of autophagy/xenophagy [214,215]. Interestingly, there is also emerging evidence of a link between the adaptation of ventilatory control mechanisms during chronic intermittent hypoxia [208,216], sleep-disordered breathing [217,218,219] and homeostatic changes to the gut microbiota, i.e., those symbiotic bacteria that provide the human host with nutrients, facilitate digestion and prevent colonisation of the gut by subversive, opportunistic pathogens [220]. Of relevance here may be the fact that the intestinal microbiota and its metabolites modulate autophagy through the mTOR pathway, while AMPK, a key regulator of autophagy, has been shown to be activated in the liver, skeletal muscle and colon of germ-free mice [221].

## 12. Clinical Perspective

From the evidence presented above, it is clear that AMPK contributes to relevant peripheral and central regulatory mechanisms governing oxygen supply and availability, which may impact upon feeding, food choice and weight gain, and vice versa, through our motivation to breathe and/or select particular nutrients. It is also evident that high-altitude adaptation may ameliorate the risk of obesity and diabetes. This raises the possibility that the selection of particular nutrients may not only lead to obesity, but also reduce oxygen demand and thus ventilatory supply, which could in turn lead to sleep-disordered breathing and pulmonary hypertension, in a manner exacerbated through consequent changes in cell-specific expression of AMPK subunit isoforms. Because our understanding of the mechanisms by which AMPK affects cardiorespiratory control and feeding is rudimentary, further research should therefore be encouraged. This may reveal new therapeutic strategies, through the modulation of the activities and/or expression of AMPK or its downstream targets. In the context of pulmonary hypertension and sleep-disordered breathing of newborns and adults, the mechanisms are largely unknown, current therapies poor, and the unmet clinical need is clear from the following statistics:

- Idiopathic neonatal pulmonary hypertension affects 60 per million, with 50%–75% survival at five years [222];
- Idiopathic pulmonary hypertension of adults affects ≈15 per million [223], where life-expectancy is ≤6 years [223];
- Sudden infant death syndrome, which has been linked to hypoventilation and apnoea of pre-term infants during hypoxia [194], is the leading cause of death in otherwise healthy infants, and currently accounts for approximately 300 deaths per annum in the UK (UK Office for National Statistics) and 2000 deaths per annum in the USA [224];
- Sleep-disordered breathing [103] affects 3–5 million in the UK (UK Office for National Statistics), 15–65 million in the USA [225] and is associated with all-cause mortality [193];
- Acute respiratory distress syndrome is associated with pulmonary hypertension [226,227], obesity and sleep-disordered breathing [228], may be opposed by AMPK [229], and is triggered by SARS, MERS [230] and COVD-19 [231].

There will likely be as yet unforeseen therapeutic potential, given that adult neuroendocrine responses to critical illness alter breathing and feeding behaviour with either beneficial or deleterious effect, depending on individual circumstance [161,232]. In this respect one final alluring fact is that previous periods of exposure to hypoxia at altitude (>8000 metres) provide up to eight weeks protection against acute mountain sickness [233], the occurrence of which is slightly higher in the obese and those with underlying lung disease, and lower in those that, irrespective of fitness, breathe more [234].
